# How machine learning is impacting research in atrial fibrillation: implications for risk prediction and future management

**DOI:** 10.1093/cvr/cvab169

**Published:** 2021-05-12

**Authors:** Ivan Olier, Sandra Ortega-Martorell, Mark Pieroni, Gregory Y H Lip

**Affiliations:** 1 School of Computer Science and Mathematics, Liverpool John Moores University, 3 Byrom Street, Liverpool L3 3AF, UK; 2 Liverpool Centre for Cardiovascular Science, Liverpool John Moores University, Liverpool, UK; 3 Liverpool Heart and Chest Hospital, Liverpool, UK

**Keywords:** Atrial fibrillation, Artificial intelligence, Machine learning, Risk analysis, Wearables

## Abstract

There has been an exponential growth of artificial intelligence (AI) and machine learning (ML) publications aimed at advancing our understanding of atrial fibrillation (AF), which has been mainly driven by the confluence of two factors: the advances in deep neural networks (DeepNNs) and the availability of large, open access databases. It is observed that most of the attention has centred on applying ML for dvsetecting AF, particularly using electrocardiograms (ECGs) as the main data modality. Nearly a third of them used DeepNNs to minimize or eliminate the need for transforming the ECGs to extract features prior to ML modelling; however, we did not observe a significant advantage in following this approach. We also found a fraction of studies using other data modalities, and others centred in aims, such as risk prediction, AF management, and others. From the clinical perspective, AI/ML can help expand the utility of AF detection and risk prediction, especially for patients with additional comorbidities. The use of AI/ML for detection and risk prediction into applications and smart mobile health (mHealth) technology would enable ‘real time’ dynamic assessments. AI/ML could also adapt to treatment changes over time, as well as incident risk factors. Incorporation of a dynamic AI/ML model into mHealth technology would facilitate ‘real time’ assessment of stroke risk, facilitating mitigation of modifiable risk factors (e.g. blood pressure control). Overall, this would lead to an improvement in clinical care for patients with AF.

## Graphical Abstract

**Figure:**
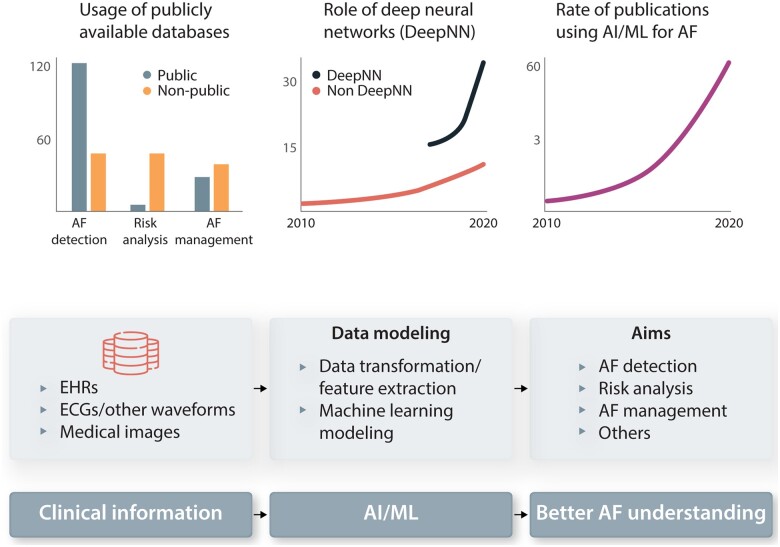

## 1. Introduction

Atrial fibrillation (AF) is the commonest arrhythmia worldwide, increasing the risk of stroke and heart failure.^1^ In the general population, diabetes mellitus (DM), high blood pressure, and coronary artery disease are regarded main risk factors. An increased risk of AF also occurs in patients undergoing major operations^2^ and those suffering from acute severe illness (e.g. infection or other pyrexical illnesses), chronic chest disease, and lifestyle factors, such as obesity.

Over the last decade, artificial intelligence (AI) has gained momentum and is rapidly becoming a mature discipline.^3^^,^^4^ The term AI was coined in the late 50 s by McCarthy, to denote the simulation of human intelligence in machines.^5^ Therefore, AI is not necessarily a newcomer, although most of its recent growth in popularity is due to machine learning (ML). ML is a branch of AI that deals with the development of algorithms that use data to make predictions and to improve their accuracy without being explicitly programmed to do so.^6^

Note that the process of learning the task of making predictions from data follows inductive logic. This means that if an ML algorithm is supplied with enough data, it should be able to provide us with an accurate response, although not necessarily 100% accurate. Unlike mechanistic models, ML models are not aimed at finding causal relations between inputs and outputs. However, they both could synergically work to accelerate the understanding of AF.^7^ In several domains, the use of the terms AI and ML are often mistakenly interchanged, sometimes accidentally, and also because of commercial reasons: AI sounds old-fashioned within some sectors. In this review, we will adhere to the definition of ML as a branch of AI.

ML has captured the interest of the medical and healthcare community, and particularly in the last 2–3 years, we have seen an explosion of publications using ML in medicine. This has also been the case in cardiovascular research, where we know ML and AI are having an impact in AF research; however, it is less known what the magnitude of such impact is. The main aim with this review is to produce a clear picture of how ML is changing the research in AF, which, ultimately, could help in gaining a better understanding of it.

## 2. Data used in this study

To conduct this study, we retrieved 465 publications from the PubMed online database that contained the terms ‘atrial fibrillation’ and either ‘machine learning’, ‘artificial intelligence’, ‘deep learning’, or combinations of them. Manuscripts were restricted to English language. Many publications were excluded as they were not directly addressing the problem of using machine learning for atrial fibrillation research. A final set of 147 publications were included in this review.

## 3. Number of publications using ML for AF is exponentially growing

**Figure 1:**
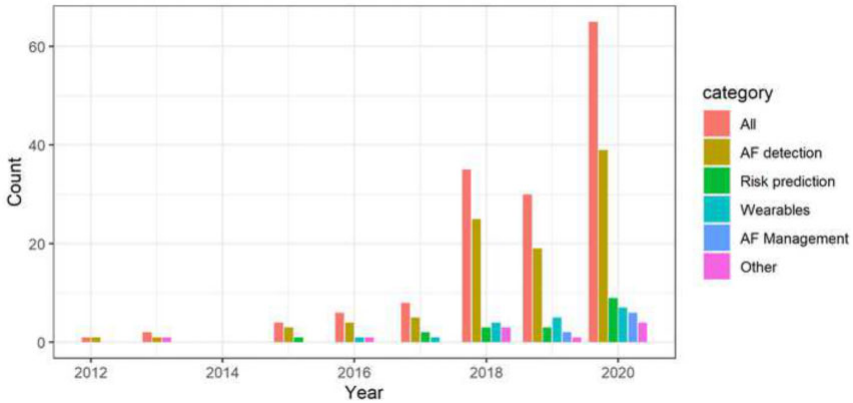
Growth in the number of ML in AF publications overall and by categories since 2012.

The use of ML in AF has attracted great attention in recent years, as evident by the ever-growing number of related scientific publications (*Figure 1*). We have identified the following non-mutually exclusive categories: AF detection, risk prediction, portable and wearable devices, management, and others. ML has been predominantly applied in AF detection, but other aspects, such as the development of ML risk prediction models and the use of wearable technology, have been of increasing interest. It is also worth highlighting the seemingly fresh interest in applying ML for AF management.

## 4. Machine learning for AF analysis

**Figure 2:**
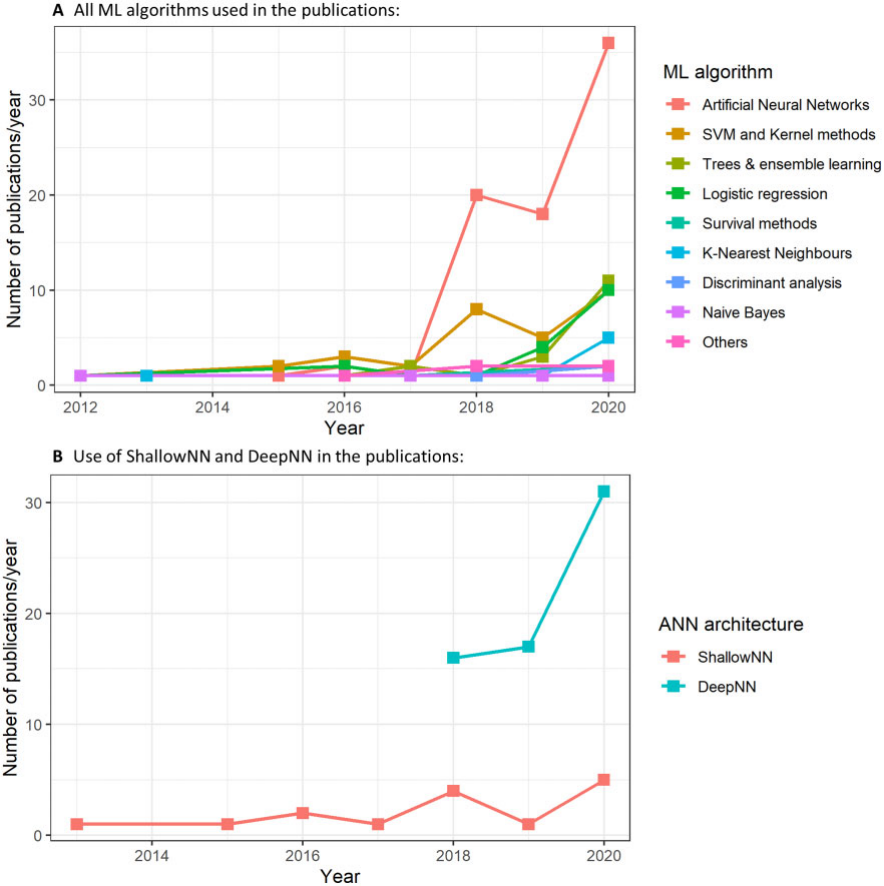
Trends of the ML algorithm families used in AF research. (*A*) All ML algorithms, with shallow and deep NNs grouped together as Artificial Neural Networks. (*B*) Separation of shallow and deep NNs.

Several ML algorithms are used for the analysis of AF. As it is seen in *Figure 2A*, artificial neural networks^8^ (ANN) have clearly become the preferred ML choice for the AF research community, particularly in the last 3 years. Within the ANN category, deep neural networks (DeepNNs) significantly outnumbered shallow neural networks (ShallowNNs), the more traditional ANNs, as can be seen in *Figure 2B*. More specialized DeepNNs, such as convolutional neural networks^8^ (CNNs) and recurrent neural networks^9^ (RNNs), are particularly popular choices. CNNs and RNNs have the key functionality of working as automatic feature extractors (i.e. that they are not pre-designed by humans), which allows them for a direct processing of data modalities commonly used for AF analysis such as electrocardiograms (ECG), echocardiograms, and cardiac magnetic resonance images (MRI).

In recent years, DeepNNs have proved to be successful in solving medical tasks at similar or higher accuracy than expert humans. However, DeepNNs have a few caveats: they typically require large amount of data to guarantee the appropriate optimization of their model parameters, and high-performance computer to reduce computing time. Furthermore, DeepNNs tend to work as ‘black boxes’, which makes difficult to explain the rationale behind their model decision making. This poses a major limitation if, instead of a predictive modelling, an explanatory analysis is required. It is worth mentioning that attempting to ‘open’ the box of DeepNN models is an active area of research.^10^^,^^11^

Other ML families use a different approach. For instance, the tree-based methods and ensemble learning family uses the combination of ‘weak’ ML algorithms, typically decision trees, as their ‘processor units’.^12^^,^^13^ Examples of them are random forest and gradient boosted machines. They have consistently shown to be excellent choices as they typically exhibit high model performance while being relatively simple to train. Tree-based ensemble learning methods can also provide some level of interpretation of the results, as opposed to ANNs. As opposite to CNN and RNN algorithms, they can only process data in tabular form. Therefore, their use for AF analysis via medical images and waveforms require the implementation of a processing stage to extract hand-crafted features before the ML modelling.

There are also algorithms, such as discriminant analysis, logistic regression, and other linear models, that could also be considered ML algorithms despite being traditionally used in statistics. In AF analysis, they are commonly used for risk prediction modelling as they offer high level of interpretability in the form of odds ratios or similar.

Attempting to delineate hard boundaries between ML families is not entirely correct since it is frequent to find algorithms that overlap across several families or share mathematical basis. There are several comprehensive reviews on ML algorithms, but we consider Deo^14^ one of the most complete as it contains most of the elements needed for an overview of ML in medicine.

**Figure 3:**
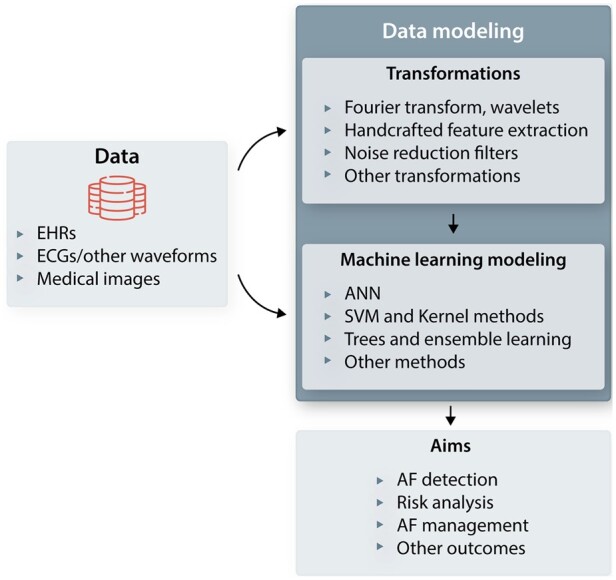
Possible ML analysis for AF. The data, as input of the analysis, could be in the form of a single or multiple modalities of electronic health records (EHRs), electrocardiograms (ECGs), and/or other waveforms, and medical images, such as cardiac MRI and echocardiograms.

*Figure 3* summarizes several ways ML is used for AF analysis. As it is seen in the figure, the format of the data could be a single modality such as electronic health records (EHRs), ECGs, or medical images (e.g. cardiac MRI), or multi-modal, when using combinations of them. The data format influences the selection of the ML algorithm as some of them, such as CNN and RNN, can process multi-modal data by design, while others require to perform some transformation to the data first. The aim of the analysis could also influence the ML algorithm choice as detecting AF is commonly defined as a prediction problem while risk analysis may involve explanatory analysis too.

## 5. Publicly available databases for AF research

**Figure 4:**
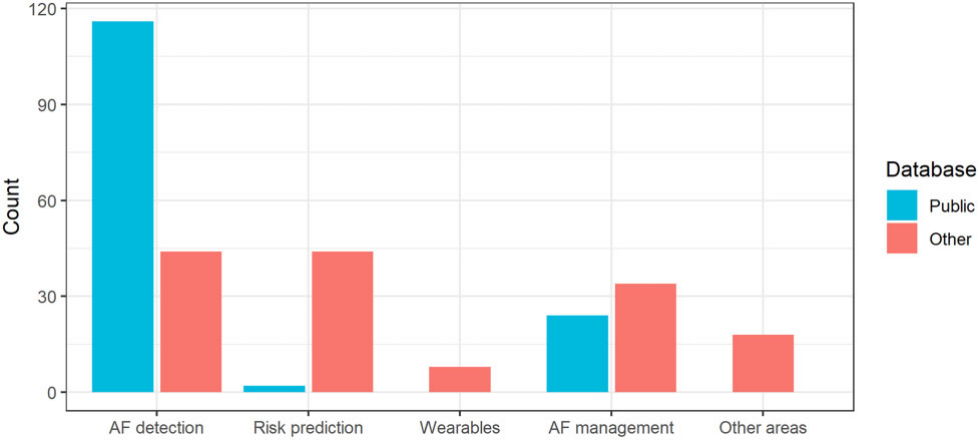
The role of publicly available databases for AF research compared to other (mainly proprietary) databases, in five AF research areas/topics: detection, risk prediction, wearables, management, and other areas.

In recent years, several databases that allow for research in AF have been made publicly available (*Figure 4*). This is likely one of the key aspects that has driven the recent interest for AF in the ML community. The modelling of AF-related data is challenging since it typically involves not only the handling of noisy multivariate time series and also the fusion of different data formats and sources.

A large number of recent publications related to ML applications in AF use at least one of these databases. They are hosted by PhysioNet^15^ (physionet.org), a large data repository for biomedical research. The MIT-BIH Atrial Fibrillation Database,^16^ which includes 25 long-term ECG recordings of human subjects with AF (mostly paroxysmal); the MIT-BIH Arrhythmia Database,^17^ which contains 48 half-hour ambulatory ECG recordings, obtained from 47 subjects; the MIT-BIH Noise Stress Test database,^18^ which includes 12 half-hour ECG recordings and 3 half-hour recordings of noise typical in ambulatory ECG recordings; and the PAF Prediction Challenge Database,^19^ which was used for the Computing in Cardiology Challenge of 2001, an open competition with the goal of developing automated methods for predicting paroxysmal AF.

Another large boost comes from the Computing in Cardiology (CinC) Challenge 2017,^20^ also organized by PhysioNet. The challenge was created to directly address the problem of identifying AF from short single-lead ECG recordings. The task was to develop a classifier to discriminate between AF, other arrhythmias, normal sinus, and noise. PhysioNet released a database with a training set with 8528 single lead ECG recordings lasting from 9 to just over 60 s and a test set with 3658 ECG recordings of similar lengths.

Other databases, such as MIMIC-III^21^^,^^22^ and UK BioBank,^23^ have also been used for AF research, although their scope is wider. MIMIC-III stands for Medical Information Mart for Intensive Care III and is a publicly available database that comprises the clinical records of more than 50 000 ICU admissions to the Beth Israel Deaconess Medical Center (MA, USA) between 2001 and 2012. In parallel, there is also available the MIMIC-III Waveform Database, which contains more than 67 000 waveforms of ∼30 000 patients, most of them also in the MIMIC-III. The UK BioBank is a very large, detailed, and prospective database that contains genetic and detailed health data of more than half a million UK participants.

**Figure 5:**
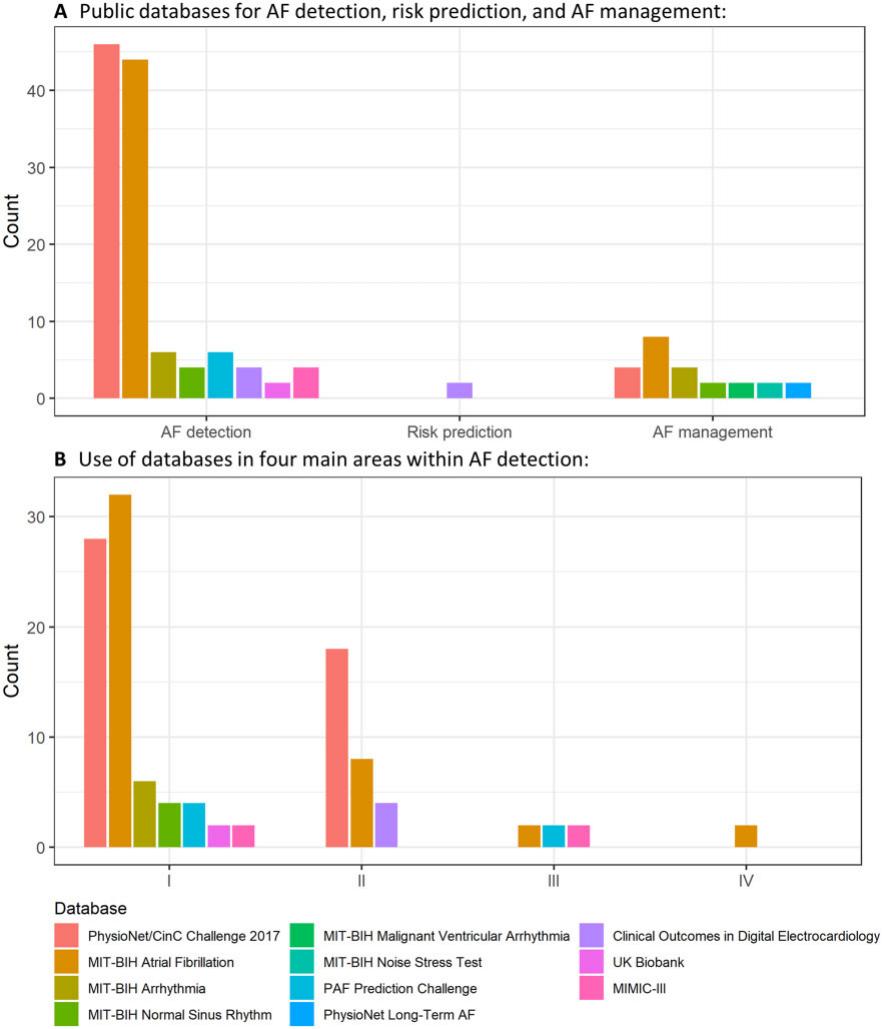
Main publicly available databases used for AF research. (*A*) Uses of these databases by AF research areas/topics. (*B*) Use of these databases for AF detection for studies that use: (i) methods that rely on transformations of the ECG, (ii) methods that require little or no transformation of the ECG, (iii) methods for the detection of new onset AF, and (iv) other approaches for AF detection using ML.

These publicly available databases have played a pivotal role in key areas of AF research, such as AF detection (*Figure 4*), where these databases have been used in numerous studies not only on their own and also to support the development of models that also use (or are validated on) other proprietary data. *Figure 5A* includes further details on the number of times these databases were used, showing that the two most popular databased have been the PhysioNet/CinC Challenge 2017 and the MIT-BIH Atrial Fibrillation databases.

*Figure 5B* shows further details on how these databases supported a variety of studies for the detection of AF using different methodological approaches, such as the use of methods that rely on transformations of the ECG, the use of methods that require little or no transformation of the ECG, methods for the detection of new-onset AF (NOAF), and other approaches for AF detection using ML. The following section will look at this in further detail.

## 6. ML for detecting AF

ML models have become very accurate in detecting AF, most of them exhibiting accuracies higher than 90%. Some models are designed to detect AF only, but there are others that also identify other arrhythmias. Data typically involve the use of ECGs, either a single or 12 leads, but there are also some methods that use other modalities, such as ballistocardiogram (BCG), photoplethysmogram (PPG), tabular data extracted from EHR, or combinations of them. Another critical question is whether transforming the data is necessary or useful before applying ML, or whether it is possible to use (almost) raw data as inputs. This decision could heavily contribute to the decision of what ML algorithm should be used. For instance, tree-based methods can handle missing values by design, CNN algorithms can directly learn from time series and/or images, etc.

### 6.1 Methods using data transformation of the ECG

Yang *et al*.^24^ were one of the first articles that used ANNs for the detection of AF in ECG signals back in 1994, specifically to separate sinus rhythm with supraventricular extrasystoles and/or ventricular extrasystoles from AF. A further model combining ANNs and deterministic logic was also implemented achieving AUC on the test sets above 0.9. Also in 1994, Cubanski *et al*.^25^ aimed at distinguishing AF from other supraventricular arrhythmias in ambulatory (Holter) ECG. More recently in 2008, Asl *et al*.^26^ proposed an algorithm based on the generalized discriminant analysis to classify the ECG recordings into six distinct categories: normal sinus rhythm, premature ventricular contraction, AF, sick sinus syndrome, ventricular fibrillation, and 2 degrees heart block. Fast forward a few years, we have seen the upsurge of publications in this area, as discussed earlier (*Figure 1*).

Various authors have extracted non-linear high order spectrum features, reporting model performances in the order of 97–98% accuracy, which could give us an indication of the expected baseline performance nowadays. More recent methodological approaches have seen the use of incremental learning models based on transfer learning in ANNs,^27^ or even the transformation of ECG waveforms into images, using only 5 beats to detect AF.^28^

Several transformations of the ECG have become widely used and essential steps in the success of AF detection as well as other arrhythmias. *Table 1* summarizes many of them along with the ML algorithms that take in the resulting features from such transformations, an extract of the data used, and the best performance reported in the different studies. As it can be observed in the table, many of them are derived from morphological characteristics of the ECG, such as RR interval—the time between QRS complexes, heart rate variability (HRV)—the variation in time between beats, and P-wave shape. They are also known as time-domain transformations. Another group of transformations work on the frequency domain, which requires the use of the Fourier transform (FT). They are useful to discriminate high vs. low frequency segments of the ECG. Transformations based on the wavelet transform^62^ (WT) apply a set of wavelets to decompose the ECG in time-frequency measurements. Wavelets are sensitive to very localized time and frequency bands. Other transformations may be used to extract statistical features such as mean and standard deviation, whilst others could be based on information theory such as entropy and distortion.

**Table 1:** Summary of publications that make use of transformations of the ECG to extract relevant features, which are then used by ML algorithms to learn how to detect AF

Study	**Transformation** ^a^	**ML algorithm** ^b^	Data	**Best performance** ^c^
Cubanski *et al*.^25^	Several morphological characteristics of the non-QRS portions of the waveforms.	ANN	47 744 ECG segments (including 32 076 AF segments and 15 668 with other supraventricular arrhythmia segments).	Se: 82.40 Sp: 96.60
Asl *et al*.^26^	Generalized discriminant analysis.	SVM	1367 ECG segments each with 32 RR intervals containing six different arrhythmia classes.	Se: 95.77 Sp: 99.40 Acc: 99.16
Mohebbi *et al*.^28^	Linear discriminant analysis on ECG.	SVM	Episodes: 1157 (835 normal episodes, 322 AF episodes). Train/test episodes for normal and AF classes were 555/280 and 214/108, respectively.	Se: 99.07 Sp: 100
Prasad *et al*.^29^	High-order spectra and independent component analysis.	KNN, ANN, DeepNN	23 ECG records, 605 episodes. A total of 641 normal, 887 atrial fibrillation, and 855 atrial flutter ECG beats were used.	Se: 98.16 Sp: 98.75 Acc: 97.65
Xia *et al*.^30^	Short-term Fourier transform and WT.	CNN	23 ECG records, 605 episodes. Only 1 ECG lead used for AF detection.	Se: 98.79 Sp: 97.87 Acc: 98.63
Xu *et al*.^31^	Combined modified frequency slice WT.	CNN	23 ECG records. 294 136 AF images + 294 136 normal images randomly selected, resulting in 588 272 images for training.	Acc: 84.85 Se: 79.05 Sp: 89.99 AUC: 0.92
Kong *et al*.^32^	Statistical features from RR intervals.	RBF, RVM	1960 patients, 10 s per lead, from which 1056 are AF patients and 904 healthy subjects.	Acc: 98.16
Lai *et al*.^33^	RR interval and F-wave frequency-domain features.	CNN	23 ECG records, segmented into 83 461 10-s ECG segments, from which 49 952 were normal and the rest were AF segments.	Se: 97.40 Sp: 97.20 Acc: 97.30
Gliner *et al*.^34^	Time-domain, frequency-domain, and statistical features.	SVM, ANN	12 186 ECG records: 60.43% normal, 0.54% noisy, 9.04% AF, and 30% other rhythm disturbances.	F1: 0.80
Sadr *et al*.^35^	Time-domain, frequency-domain, and statistical features.	ANN	12 186 ECG records: 8528 for training and 3658 for test.	F1: 0.78
Shi *et al*.^27^	The waveform and some statistics of the RR interval (mean, standard deviation, entropy).	ANN	48 ECG records (2 leads) with 30 min duration, and 25 long-term (c. 10 h) ECG recordings from AF patients, also 2 leads.	Acc: 97.53 Se: 100
Liu *et al*.^36^	P-wave absence detection, statistical, information theory, and frequency domain features.	SVM	12 186 ECG records: 8528 for training and 3658 for test.	F1: 80
Andersen *et al*.^37^	Time-domain features.	SVM, DeepNN	12 186 ECG records: 8528 for training and 3658 for test.	Se: 96.81 Sp: 96.20 AUC: 0.99
Asgari *et al*.^38^	Stationary WT.	SVM	12 186 ECG records: 8528 for training and 3658 for test.	Se: 97 Sp: 97.1 Acc: 97.1 AUC: 99.95
Xin *et al*.^39^	Wavelet multi-scale entropy features of HRV.	SVM	23 ECG records, 605 episodes.	Se: 94.88 Sp: 89.48 Acc: 92.18
He *et al*.^40^	ECG waveforms transformed into images using WT	CNN	23 ECG records, 605 episodes.	Se: 99.41 Sp: 98.91 Acc: 99.23
Lown *et al*.^41^	De-correlated Lorenz plots of 60 consecutive RR intervals, followed by WT to compress the resulting images.	SVM	ECG records: 250 h from 25 subjects and 24 h of data from 47 subjects. Validation data: 415 subjects, 79 with AF, and 336 without.	Se: 100 Sp: 97.6
Hernandez *et al*.^42^	WT, time-domain, and frequency-domain features.	FCNN	12 186 ECG records: 8528 for training and 3658 for test.	Se: 95.70 Sp: 72.39 F1: 64
Wu *et al*.^43^	WT-based features	CNN	For all the 17 850 ECG segments, 60% for training, rest for test.	Acc: 97.56 Se: 97.56 Sp: 99.19 AUC: 99.83
Herraiz *et al*.^44^	Transformed ECGs into scalograms	CNN	Samples from available data sources: 1000 + 500 ECG records. From a proprietary database: 1000 ECG records.	Se: 94.42 Sp: 90.61 Acc: 92.51
Hong *et al*.^45^	Hand-crafted features based on medical domain knowledge, and CNN-based features.	Gradient boosting decision trees	12 186 ECG records: 8528 for training and 3658 for test.	F1: 82.5
Smisek *et al*.^46^	Time-domain features.	SVM	12 186 ECG records: 8528 for training and 3658 for test.	F1: 81
Sodmann *et al*.^47^	CNN-based features.	GBM	12 186 ECG records: 8528 for training and 3658 for test. 12 million characteristic waveforms were used as input volume. The assigned annotation codes of each segment’s midpoint peak were used as output volume.	F1: 82
Rubin *et al*.^48^	Noise reduction filter followed by WT.	CNN	12 186 ECG records: 8528 for training and 3658 for test. Additional 30-s ECG segments (6312 records) with AF were collected from various sources to augment the training and validation sets.	F1: 82
Khamis *et al*.^49^	Artefact masking filters and QRS detection algorithms followed by RR intervals, PQRST morphologic, and artefact/noise ratio features.	FCNN, ensemble learning	12 186 ECG records: 8528 for training and 3658 for test.	F1: 80
Bashar *et al*.^50^	HRV-derived density Poincaré plots followed by image processing.	KNN, SVM, and RF	ECG recordings obtained from 20 subjects, resulting in a total of 500 AF and 340 PAC/PVC segments. Seven additional subjects (2 with persistent AF, 5 had PAC/PVC rhythms).	Se: 98.99 Sp: 95.18 Acc: 97.45
Oster *et al*.^51^	HRV-derived density Poincaré plots and morphologic features.	SVM, DeepNN	450 subjects from the UK BioBank dataset. Expert annotations in this study classified 52 subjects with AF out of 450.	F1: 84.8 Se: 75
Jalali *et al*.^52^	2D-ECG spectrogram features generated from short-term Fourier transforms.	CNN	ECG records from various publicly available data sources: 25 AF and 25 normal rhythms, each containing one 30-min ECG segment; 23 annotated ECG records from a Holter monitor of AF patients; and 8528 short ECG recordings.	Se: 99.9 Sp: 99.7 Acc: 99.8
Ebrahimzadeh *et al*.^53^	Time-domain, frequency-domain, and non-linear analysis of HRV.	Mixture of experts	106 signals from 53 pairs of 30-min ECG recordings, one ECG segment before PAF onset and another one at least 45 min distant from the onset.	Se: 100 Sp: 95.55 Acc: 98.21
Marinucci *et al*.^54^	Several morphological, F-waves, and HRV features.	FCNN	8028 ECG records (training: 4493; validation: 1125; testing: 2410) classified into AF and non-AF cases.	Se: 81.2 Sp: 81.2 AUC: 90.38
Boon *et al*.^55^	Time-domain, frequency-domain, and non-linear analysis of HRV.	SVM	106 signals from 53 pairs of 30-min ECG recordings, one ECG segment before PAF onset and another one at least 45 min distant from the onset.	Se: 86.8 Sp: 88.7 Acc: 87.7
Baalman *et al*.^56^	Morphological features.	DeepNN	1469 ECG records from participants in the AF Ablation and Autonomic Modulation via Thoracoscopic Surgery (AFACT) trial.	Acc: 96 AUC: 97 F1: 94
Abdul-Kadir *et al*.^57^	Second order dynamic system-based features.	ANN, SVM	41 ECG records from two publicly available data sources.	Acc: 95.3
Ghosh *et al*.^58^	Multi-rate cosine filters.	DeepNN	c. 71 ECG records from various publicly available data sources. Different data combinations trialled.	Acc: 94.40 Se: 98.77 Sp: 100
Kisohara *et al*.^59^	Heartbeat interval Lorentz plots imaging of different segment window lengths.	CNN	LP images of non-overlapping segments (10–500 beats length) were created from 24-h ECG RR intervals in 52 patients with chronic AF and 58 non-AF controls as training data and in 53 patients with PAF and 52 non-AF controls as test data.	Acc: 97.9 AUC: 98.7
Iqbal *et al*.^60^	Time-domain and frequency-domain features	DeepNN	More than 36 ECG records, including 10 subjects of flattened T waves, 20 of normal sinus rhythm, and 6 AF subjects.	Acc: 99.99
Buscema *et al*.^61^	RR intervals and time window composition-based features.	SCM	73 ECG records, 33 of them with AF annotations, and other 31 with a different pathological annotation.	F1: 95.16 Se: 96.34 Sp: 92.80 Acc: 94.99

a Transformation: WT, wavelet transform.

b ML algorithms: ANN, artificial neural networks; SVM, support vector machines; KNN, k-nearest neighbor; DT, decision trees; CNN, convolutional neural networks; RBF, radial basis functions; RVM, relevance vector machine; DeepNN, deep neural networks; FCNN, fully connected neural networks; GBM, gradient boosted machines; RF, random forest.

c Performance metrics: Se, sensitivity; Sp, specificity; Acc, accuracy; F1, F1-score; AUC, area under the operator receiver curve (ROC).

A large proportion of studies used the PhysioNet/CinC 2017 Challenge^34–36^^,^^42–46^^,^^48^^,^^49^ and the MIT-BIH Atrial Fibrillation Database,^27^^,^^31–33^^,^^37–41^^,^^63^ making them the two data sources most used to detect AF. Waveforms from the MIMIC-III database were used by Bashar *et al*.^50^ to train an ML model to detect AF, while using a wearable armband ECG dataset and the PhysioNet MIT-BIH Atrial Fibrillation Database for test. The UK Biobank dataset was used by Oster *et al*.,^51^ while Jalali *et al*.^52^ used the Keimyung University Dongsan Medical Center dataset and the public datasets PAF Prediction Challenge Database, MIT-BIH Atrial Fibrillation Database, and PhysioNet/CinC Challenge 2017. These are a few examples where publicly available datasets have been used to support the development of models to detect AF that have been later tested and/or validated on in-house datasets. Other studies^54–56^ used less known, more specific and/or restricted access databases.

There are several publications where new ML algorithms or variants of existing ones were proposed. For instance, Abdul-Kadir *et al*.^57^ used a second-order dynamic system to extract features form ECG recordings; Ghosh *et al*.^58^ extracted features from single-lead ECG recordings using a multi-rate cosine filter bank architecture for the evaluation of coefficients from the ECG signal at different sub-bands; a DeepNN algorithm known as Hierarchical Extreme Learning Machine used the extracted features to detect AF; and Kisohara *et al*.^59^ assessed the performance of heartbeat interval Lorenz plot (LP) imaging for AF detection, using the resulting images as inputs of the ML algorithms.

### 6.2 Methods requiring little or no transformation of the ECG

*Table 2* shows a summary of the studies that implemented ML models to detect AF requiring little or no transformation of the ECG recordings. As mentioned above, this kind of models works directly with the ECG as input and use either CNN or RNN to automatically extract data features as part of the pipeline of detecting AF.

**Table 2:** Summary of publications that use ML algorithms to detect AF requiring little or no transformation of the ECG

Study	ML algorithm	Data	Best performance
Faust *et al*.^64^	DeepNN, LSTM	23 ECG records from different subjects, 10 h each, containing two ECG signals with AF annotations.	Acc: 99.77 Se: 99.87 Sp: 99.61 AUC: 100
Erdenebayar *et al*.^65^	CNN	19 804 short-term ECG segments were extracted from 139 subjects: 11 882 AF segments and 7922 normal segments.	Acc: 98.7 Se: 98.7 Sp: 98.6 AUC: 100
Kamaleswaran *et al*.^66^	CNN	12 186 ECG records: 8528 for training and 3658 for test.	F1: 0.83
Hsieh *et al*.^67^	CNN	10 151 ECG samples: 903 AF, 5959 normal, 299 noisy, and 2990 other.	F1: 78.2 Acc: 80.8
Ping *et al*.^68^	CNN, LSTM	12 186 ECG records: 8528 for training and 3658 for test	F1: 89.55 Se: 87.42 Sp: 91.37 Acc: 85.06
Warrick *et al*.^69^	CNN, LSTM	12 186 ECG records: 8528 for training and 3658 for test	F1: 82
Xiong *et al*.^70^	CNN, RNN	12186 ECG records: 8528 for training and 3658 for test	F1: 82
Parvaneh *et al*.^71^	CNN	12 186 ECG records: 8528 for training and 3658 for test. An additional 6312 ECG segments with AF from various sources were used when training. A total of 18 498 records were used collectively with 3658 used for validation.	F1: 82
Ribeiro *et al*.^72^	CNN	12 lead ECG records from 1 558 415 patients.	F1: 80 Sp: 99
Ribeiro *et al*.^73^	CNN	12 lead ECG records from 1 676 384 patients.	F1: 80 Sp: 99
Tran *et al*.^74^	DeepNN	12 186 ECG records: 8528 for training and 3658 for test	F1: 80 AUC: 85
Plesinger *et al*.^75^	CNN, Ensemble learning	12 186 ECG records: 345 removed due to disagreement with expert labelling, 8183 used for training and 3658 for test.	F1: 83
Cai *et al*.^76^	FCNN	16 557 samples of 12-lead ECG recordings from 11 994 subjects: 3353 AF, 5650 normal, and 7554 other abnormalities.	Acc: 99.35 Se: 99.19 Sp: 99.44
Fan et at. ^77^	CNN	12 186 ECG records: 8528 for training and 3658 for test	Acc: 98.13 Se: 93.77 Sp: 98.77
Lee *et al*.^77^	CNN	20 000 unique participants: 10 000 normal sinus rhythm and 10 000 AF.	Acc: 99.90
Mousavi *et al*.^78^		162 536 5-s segments were extracted from 25 long-term ECG records: 61 924 AF segments, and 100 612 non-AF segments.	Se: 99.53 Sp: 99.26 Acc: 99.40
Lai *et al*.^79^	CNN	Long-duration ECGs recorded from patch-based leads. More than 510k 10-s ECG segments were extracted.	Acc: 93.1 Se: 93.1 Sp: 93.4
Mousavi *et al*.^80^	RNN	162 536 5-s segments extracted from 25 long-term ECG records. 12 186 additional ECGs were used from publicly available datasets.	Se: 99.08 Sp: 98.54 Acc: 98.81 AUC: 99.86
Zhang *et al*.^81^	CNN	277 807 12-lead static ECG records lasting 10–60 s.	Acc: 98.27 Se: 99.95
Attlia *et al*.^82^	CNN	A single 10-s, 12-lead ECG was acquired during normal sinus rhythm from 180 922 patients.	AUC: 90 Se: 82.3 Sp: 83.4

Please refer to *Table 1* for other acronyms.

LSTM, long short-term memory.

The MIT-BIH Atrial Fibrillation Database was used by Faust *et al*.^64^ which implemented a two-stage DeepNN model, first, training to detect RR intervals, and second, an LSTM model that used the ECG segments. The PhysioNet/CinC Challenge database was used several studies.^66–71^^,^^74^^,^^75^ Other databases were also used for AF detection with little or no transformation of the ECG, e.g. Ribeiro *et al*.^72^^,^^73^ used a very large database named Clinical Outcomes in Digital Electrocardiology. Tran *et al*.^74^ implemented a multiplicative fusion of two DeepNN models, one of the single models using hand-crafted features while the other one, the raw ECG recordings, with authors claiming that the fusion model outperformed the single models when analysed individually; and Plesinger *et al*.^75^ which implemented two ML algorithms to be used in parallel, one of them a CNN model that processed the raw ECG, the second one an ensemble learning algorithm that received several hand-crafted features, both algorithms attempted to predict the classes, and the final decision was made based on prediction certainty.

Novel ML architectures have also been proposed. For instance, Fan *et al*.^83^ proposed a multi-scaled fusion of CNNs that employs two streams of CNNs to capture features of different scales, where the learned features were visualized and compared against linear methods; Lee *et al*.^77^ implemented and evaluated up to 30 different CNN architectures; Mousavi *et al*.^78^ implemented a two-channel CNN model: the first one aimed to identify where to look for the detection of AF in the ECG, while the second one to perform the actual AF detection; Mousavi *et al*.^80^ developed an interpretable RNN for AF detection, and claimed that the model was able to explain the reasons behind their decisions whilst still retaining performance.

An interesting test was performed by Attlia *et al*.^82^ which consisted in assessing the feasibility of accurately detecting AF using a single 10-s, 12-lead ECG was acquired during normal sinus rhythm. AF signature was found using a CNN model that exhibited performance levels that could allow for its use in clinical settings. Their model achieved even higher performance if repeated ECGs were used over a month time window.

### 6.3 NOAF detection

A smaller proportion of the studies concentrated on NOAF. Boon *et al*.^84^ investigated the effect of 15- and 30-min segments of HRV prior to NOAF, using for this extracted statistical features on an SVM model. Chesnokov *et al*.^85^ attempted a more distant prediction by analysing changes in the HRV dynamics and showed satisfactory result predicting paroxysmal AF up to 60 min before the event. Their ANN and SVM models were trained on extracted features using spectral and complexity analysis. Tse *et al*.^86^ developed a decision tree model for NOAF in mitral stenosis based on features extracted from the ECG, plus several clinical and demographic factors (e.g. age and systolic blood pressure), while Bashar *et al*.^87^ proposed an ML algorithm for NOAF detection during sepsis using data extracted from the MIMIC-III database.

### 6.4 Other approaches for AF detection using ML

There have been other approaches used for AF detection that are less related to the previous categories mentioned. Zalabarria *et al*.^88^ proposed an AF diagnosis algorithm based on ANNs that uses parameters extracted from short-length heart period measures obtained by arterial pulse wave foot point detection, while Yan *et al*.^89^ used video and a pretrained CNNs to analyse facial PPG signals in AF detection. Two other studies^90^^,^^91^ used electronic health records (EHR): in the case of Karnik *et al*.,^90^ the authors implemented several ML algorithms to predict AF and atrial flutter but model performances were considerable low in comparison to ECG or other waveforms counterparts, as expected. However, the authors argued that the study has its merits as it could identify AF risk factors. In the case of Tiwari *et al*.,^91^ ∼200 common EHR features, such as age, sex, past clinical history, and vitals, were used to predict AF using an FCNN. The model was compared against a multiple logistic regression model showing non-significant improvement in performance.

Other less common approaches also include the development of an ML model to predict future AF among patients with no history of AF, by Christopoulos *et al*.,^92^ with results independently corroborated using Cox regression. Chua *et al*.^93^ used circulating blood-based biomarkers along with clinical and demographic features to predict undetected AF. Jo *et al*.^94^ proposed a DeepNN model based on variational autoencoders that predicts AF highly accurately and provides some model interpretability. Da Poian *et al*.^95^ used compressive sensing approaches to ECG, which is a signal processing technique that exploits signal sparsity to reconstruct it, and conclude that compressing the signals still produces comparable results to features extracted from QRS, but can make the modelling process significantly faster.

## 7. Risk prediction modelling with AI/ML methods

A variety of risk prediction models have been developed using AI/ML methods. Some of them related to the risk of developing AF, as it is the case of Censi *et al*.,^96^ which produced a model to quantify morphological aspects of the P-wave to improve the identification of patients having different risks of developing AF. Another example is the study from Suzuki *et al*.,^97^ where they developed a model that was able to identify non-valvular AF with high performance. Non-valvular AF is associated with an increased risk of stroke; however, many patients are diagnosed after onset.

Several studies concentrated on predicting the risk of AF recurrence. In the study by Budzianowski *et al*.,^98^ the focus was on identifying the laboratory and clinical parameters responsible for early recurrence of AF following cryoballoon ablation. Bhalodia *et al*.^99^ also proposed a method that deals with AF recurrence prediction, this time using statistical shape modelling techniques on left atrium MRI scans.

Shade *et al*.^100^ developed a model to predict which patients are more likely to experience AF recurrence after pulmonary vein isolation (PVI), using pre-PVI late gadolinium-enhanced MRI scans, while Liu *et al*.^101^ proposed a model using pre-ablation pulmonary vein computed tomography to predict the trigger origins in patients with paroxysmal AF receiving catheter ablation, aiming at identifying patients with a high risk of non-pulmonary vein trigger before ablation, to reduce the recurrence of post-ablation AF.

Tse *et al*.^102^ aimed at improving the risk stratification for adverse outcomes in heart failure, such as incident AF, transient ischaemic attack (TIA)/stroke, and all-cause mortality, while Wu *et al*.^103^ focused on a more specific risk stratification model of young patients with hypertension. Hospital readmissions data for AF patients undergoing catheter ablation was investigated by Hung *et al*., to estimate the risk factors behind 90-^104^ and 30-day^105^ hospital readmissions.

The risk of mortality associated with the presence of AF was evaluated in Ribeiro *et al*.,^72^ showing that AF was a strong predictor of cardiovascular mortality and mortality for all causes, with increased risk in women. Additional cardiovascular outcomes were evaluated in Ambale-Venkatesh *et al*.,^106^ including all-cause mortality, stroke, coronary heart disease, and all atherosclerotic cardiovascular disease combined outcomes, incident heart failure, and AF.

Several articles considered the way AF increases the risk of ischaemic stroke and other thromboembolisms. Some examples are Han *et al*.^107^ studied how AF severity or burden can further risk stratify stroke patients, particularly for near-term events, while Li *et al*.^108^ worked on improving prediction models that would help identify risk factors for thromboembolism. In a more recent study, Li *et al*.^109^ proposed a model to be used especially when typical risk factors are unknown to improve stroke screening efficiency, while Kamel *et al*.^110^ studied the associations between cardioembolic stroke and AF. A study from Akça *et al*.^111^ aimed at identifying sex-specific risk factors, investigating the risk factors of post-coronary artery bypass grafting AF in patients without history of AF, while Bundy *et al*.^112^ developed models with the aim of improving the prediction of 5-year AF risk.

Goto *et al*.^113^ developed a model for predicting clinical outcomes, such as major bleeding, stroke/systemic embolism, and death, in newly diagnosed AF patients who were treated with vitamin K antagonists, using serial prothrombin time international normalized ratio values collected within 1 month after starting treatment. In a different article, Feeny *et al*.^114^ researched whether ML models could predict echocardiographic cardiac resynchronization therapy beyond current guidelines, and found that it was possible, although there is still room for improvement in this area.

Xiong *et al*.^115^ performed meta-analysis to investigate the association between DM and NOAF, obtaining that patients with DM had 49% greater risk of developing AF compared with individuals without DM. After adjusting for three additional risk factors, i.e. hypertension, obesity, and heart disease, the relative risk reported was 23%.

## 8. AI/ML in AF management

In some cases, AI/ML models have been used for predicting or understanding factors related to the management of AF patients, e.g. drug dosing, success of certain procedure or treatment, etc. Some examples have been chosen below, although many of the risk prediction studies mentioned above would also inform AF patients’ management.

The initiation of the antiarrhythmic medication dofetilide requires 3 days of telemetry monitoring due to heightened risk of toxicity within this period, and there is a range of approaches to dosing the medication. Levy *et al*.^116^ proposed the use of reinforcement learning for evaluating dose adjustment decisions, attaining an accuracy of 96%, and found that making dose adjustments, particularly at later time points, was associated with less probability of successful initiation of the medication. The authors argued that this finding could reduce healthcare costs, as it would, for example, save time and money to stop the initiation process early in a patient in whom the probability of successful initiation is unlikely.

The study from Vinter *et al*.^117^ attempted to improve the understanding of which patients would benefit from electrical cardioversion, which is frequently performed to restore sinus rhythm in patients with persistent AF. However, AF recurs in many patients and identifying those who benefit from electrical cardioversion remains challenging in clinical practice. The study was conducted in women and men separately, using logistic regression and random forest to develop sex-specific prediction models for successful cardioversion. The results presented showed modest predictive performance for successful electrical cardioversion, with best reported results being 60% accuracy for women and 59% for men.

Another study proposed by Alhusseini *et al*.^118^ focused on improving the mapping of intracardiac activation in AF using CNN, with 95% accuracy on a separate test set. They also used explainability analyses (applying gradient-weighted class activation mapping) to show that results agree with experts, which may provide immediate clinical utility to guide ablation. The study from Ghrissi *et al*.^119^ resulted in a model to automatically identify ablation sites based on their spatiotemporal dispersion, which is the delay of the cardiac activation observed in intracardiac electrograms across contiguous leads. The performance of the best model exhibited a 90% accuracy, which was obtained when using a CNN inspired architecture on augmented data. The aim was to use this model to aid patient-tailored catheter ablation procedures for treating persistent AF.

## 9. Portable and wearable devices

PPG monitoring has been implemented in many portable and wearable devices. Its simplicity and cost-effectiveness have facilitated its daily use for health and fitness tracking, enabling continuous monitoring of cardiac rhythm.^120^ Numerous studies^41^^,^^44^^,^^46^^,^^54^^,^^121–126^ have successfully used PPG for AF detection, several of them using DeepNN models.

Some artefacts in PPG signals can lead to missed episodes, which can be a limitation in some scenarios such as the detection of paroxysmal AF. Different studies^44^^,^^120^^,^^125^^,^^127^ have centred the efforts on dealing with this issue, proposing approaches to assess the quality of the signals in the presence of AF. For example, Torres-Soto *et al*.^125^ used an unsupervised transfer learning CNN autoencoder to filter noise out from the PPG signals. Other studies evaluate the quality of the signals in wearable devices, such as Sadrawi *et al*.,^128^ where quality is evaluated against the ANSI/AAMI EC57:2012 standard.

Wasserlauf *et al*.^129^ showed that an AF-sensing watch was highly sensitive for detection of AF and assessment of AF duration in an ambulatory population, when compared with simultaneous recordings from an insertable cardiac monitor. Also using a standard smartphone, this one equipped with Google Android OS, Lahdenoja *et al*.^130^ intended to detect AF via the use of the accelerometer and gyroscope.

Other studies^131^^,^^132^ have proposed the use of ML on BCG recording during sleep, reporting accuracies above 90% and arguing that BCG could be used to detect AF in home-monitoring applications. A contrasting study by Kido *et al*.^133^ focused on making the use of capacitive ECG a viable option for heart monitoring (measuring the cardiac electrical signal via capacitive coupling between electrodes and skin). The results obtained using CNNs were encouraging, although it was reported that the instability in the quality of the signal hinders its further use.

Remote-monitoring data from patients with cardiac implantable electronic devices have also been used. Han *et al*.^107^ used it to predict risk of stroke, while Lai *et al*.^79^ showed how a patch-based ECG lead, together with DeepNN-based algorithms, could provide an accurate and inexpensive tool for AF mass screening. Publicly available databases of ambulatory ECG have also been widely used,^33^^,^^41^^,^^54^^,^^60^^,^^128^ playing a substantial role in the methodological advances in this area.

## 10. Other perspectives

This section comprises a selection of other AF studies, not specifically related to AF detection, risk prediction models or AF management. They would cover subjects such as localization of AF drivers, segmentation of the left atrium, and impact of pollution on cardiovascular systems.

McGillivray *et al*.^134^ proposed a method to locate re-entrant drivers using a collection of indirect electrogram measurements. The method successfully located drivers in tissues containing a single driver of AF, as well as in tissues containing two drivers, although in its current form, the presented techniques are not refined enough to be used in clinical settings.

A more recent study on AF drivers by Zolotarev *et al*.^135^ uses ML to model electrogram frequency spectra, aiming to accurately automate driver detection by multielectrode mapping and add some objectivity to the interpretation of multielectrode-mapping findings, since AF driver detection by clinical surface-only multielectrode mapping has relied on subjective interpretation of activation maps. The developed model was competitive, but further work will be needed to increase performance.

Zahid *et al*.^136^ produced a model that shows that AF in fibrotic substrates is perpetuated by re-entrant drivers persisting in fibrosis boundary zones characterized by specific regional fibrosis metrics. The results reported provide new insights into the mechanisms that sustain persistent AF and could pave the way for personalized management of the condition.

Some studies have centred on the segmentation of the left atrium. For example, in 2018 Jin *et al*.^137^ presented an approach for the segmentation and quantitative assisted diagnosis of AF using 4D computed tomography data. The experimental results showed that this approach could construct the 3D left atrial appendage geometries. Later in the year, the authors published another study^138^ using a more robust methodological approach for this segmentation.

In 2019, Xiong *et al*.^139^ proposed a model to automatically segment late 3D gadolinium-enhanced MRI of the left atrial epicardium and endocardium on AF patients, indicating to have outperformed other state-of-the-art methods, having tested against the largest known dataset for left atrial segmentation. Later in 2020, Du *et al*.^140^ also proposed an approach for segmentation and visualization of the left atrium using the same kind of images. The authors reported to have outperformed other state-of-the-art methods and suggested this method could improve the clinical diagnosis and treatment of AF.

Recently, two studies^141^^,^^142^ paid attention to the influence of air pollution on cardiovascular systems. Yang *et al*.^141^ examined the impact of fine particulate matter pollution on the cardiovascular system and found that ambient exposure to them was linked with increased risk of arrhythmias in outpatients visiting Shanghai community hospitals, with an immediate or lag effect. Kim *et al*.^142^ also found results suggesting such associations and used them to predict incident AF.

## 11. Discussion

This review has highlighted the exponential growth of publications using AI/ML in AF research in the recent years. They are advancing our understanding of atrial fibrillation, broadly in relation to the following categories: AF detection, risk prediction, portable and wearable devices, management, and others.

Precise comparisons between reported results are not feasible as factors, such as data sources, task specificities, and error metrics, would greatly affect the performance scores. However, we observed that most of the studies modelling the task of detecting AF with ML-reported model performance that suggests that ML could fail to detect AF in between 1 in 10 and 1 in 100 of the cases, particularly if ECGs are used as data format. This could suggest that a natural ceiling might have been reached already in what is possible to achieve with this specific task and data format. However, by no means this is an indication that research in AF detection with ML is finalized, but a suggestion that perhaps the attention should move to other related questions, such as the early AF detection as investigated by Attlia *et al*.^82^ We also found that other data modalities are significantly less used, which could be associated with clinical needs and costs. However, we consider there is clinical value in combining modalities in the analysis of AF which could be helpful to improve the performance of the models, and/or to discover new features or biomarkers.

From the clinical perspective, AI/ML can help expand the utility of AF detection and risk prediction especially for patients with additional comorbidities. What are the appropriate measures to operationalize this? The use of AI/ML for detection (especially with the growth of portable and wearable devices) and risk prediction into Apps and smart mHealth technology would enable ‘real time’ dynamic assessments, incorporated into patient management pathways. As an illustrative example, the AF patient pathway could perhaps apply risk reassessment(s) at intervals, when not on antithrombotic therapy (e.g. when newly diagnosed), and while on aspirin (e.g. with background vascular disease) and post-anticoagulation (whether on warfarin or direct oral anticoagulants). AI/ML could adapt to these treatment changes over time, as well as incident risk factors. The latter can then be proactively management.

Some of the potential opportunities here are illustrated by the mHealth technology to improve optimization of integrated care in patients with Atrial Fibrillation App programme (mAFA) which investigated mHealth technology for improved screening and integrated care in patients with AF, facilitating early diagnosis, dynamic (re)assessments of risk profiles, and holistic AF management.^143^ In the prospective cluster randomized clinical trial, this integrated care approach significantly reduced the composite outcome of ‘ischaemic stroke/systemic thromboembolism, death, and rehospitalization’ compared with usual care,^144^ with long-term adherence of >70% and high (>90%) persistence of use.^145^ Such use of mHealth opportunities to improve holistic care (detection, ‘real time’; risk assessment, management optimization, and patient empowerment) has the potential to improve outcomes, especially if patients have good adherence and persistence with the approach (as shown in the mAFA trial long term extension).^145^ Ongoing studies are likely to address these issues in UK and EU countries.

In conclusion, incorporation of a dynamic AI/ML model into mHealth technology would facilitate ‘real time’ assessment of stroke risk, facilitating mitigation of modifiable risk factors (e.g. blood pressure control). Overall, we feel that this would lead to an improvement in clinical care for patients with AF.

##  

**Conflict of interest:** none declared.

## Funding

This work was partially funded by the LJMU FET Scholarship 2019.

## Notes

This article is part of the Spotlight Issue on Atrial Fibrillation.
